# The feasibility of a patient oriented interactive panoramic virtual tour for external beam radiation therapy

**DOI:** 10.3389/fonc.2025.1568405

**Published:** 2025-05-30

**Authors:** Joseph B. Schulz, Piotr Dubrowski, Xi Ling, Yufan Wu, Yushen Qian, Lynn Million, Carol M. Marquez, Amy Yu

**Affiliations:** Department of Radiation Oncology, School of Medicine, Stanford University, Stanford, CA, United States

**Keywords:** patient education, virtual reality, patient navigation, radiation therapy (radiotherapy), radiation oncology, virtual tour, patient experience

## Abstract

**Introduction:**

The integration of digital technology in healthcare, particularly for patient education and experience, is rapidly advancing. This pilot study examined the feasibility of an interactive panoramic virtual tour for improving patient experiences in an external beam radiation therapy (RT) setting at a smaller center.

**Methods:**

A virtual tour of the RT department was developed using specialized software and 360-degree 8K camera. The study utilized a two-group design: a control group (33 patients) not exposed to the tour and an experimental group (35 patients) who accessed the tour via the MyHealth platform prior to RT treatment. The survey measured levels of anxiety, comfort with treatment course, knowledge about the facility, navigation through a course of RT, and satisfaction with overall treatment on a 1–10 scale, with 10 being a more desirable outcome.

**Results:**

The experimental group reported improved outcomes compared to the control group across all parameters: anxiety levels were lower (mean: 7.3 ± SD: 2.6 vs. 6.5 ± 3.3; p = 0.32), though variances differed significantly (p = 0.03). Comfort levels were higher (9.1 ± 1.7 vs. 8.4 ± 2.1; p = 0.27), knowledge about the cancer center increased (8.7 ± 1.5 vs. 7.8 ± 2.4; p = 0.27) with unequal variances (p = 0.03), and ease of navigation slightly improved (9.8 ± 0.6 vs. 9.4 ± 1.9; p = 0.61). Satisfaction levels were similar in the experimental group (9.6 ± 1.1 vs. 9.5 ± 1.2; p = 0.74).

**Conclusions:**

This pilot study provides preliminary evidence that an interactive virtual tour may enhance certain aspects of the RT patient experience, although the small sample size limits the ability to draw definitive conclusions. The integration of virtual tours into RT practices reflects a shift towards more interactive and patient-friendly approaches in healthcare by demystifying the RT process and providing accessible information. Future research with larger, more diverse cohorts at a larger institution is warranted to confirm whether these early findings generalize more broadly and to better quantify the impact of virtual tours on patient-reported outcomes.

## Introduction

In the rapidly evolving landscape of healthcare, technology is playing a transformative role, particularly in the domain of patient education and experience (1, 2). The introduction of virtual tours for medical procedures, notably external beam radiation therapy (EBRT), is a prime example of this trend (3–5). EBRT often involves a setting unfamiliar to patients. The daunting machinery and its sounds can instigate anxiety related to the radiation-related procedure, a factor that can deter patients from pursuing or adhering to treatment. Research has consistently demonstrated that patient anxiety can have adverse effects on treatment success, adherence, and overall satisfaction (2, 6–8). This is where the potential of virtual tours becomes evident.

The advent of COVID-19 has further underscored the necessity for innovative approaches in healthcare, particularly those that address patient anxiety while upholding the standard of care (9). Digital technology, particularly immersive virtual tours akin to Google Street View, empowers patients to acquaint themselves with the treatment environment at their own pace. By providing a virtual walkthrough of the treatment facilities and treatment delivery systems, these tours can play a crucial role in demystifying the process and alleviating fears associated with it, with potential to improve the patient experience. However, with the increasing volume of educational materials available to patients, there arises a challenge: the risk of information overload (10). In this context, a centralized, interactive platform that aligns seamlessly with the natural care pathway can be invaluable. Such a platform would not only consolidate pertinent information but also facilitate easy navigation, thus enhancing the utility and accessibility of these resources. Moreover, the interactive nature of virtual tours is a critical aspect. Unlike passive information sources, an interactive tour allows patients to engage actively with the content, fostering a deeper understanding and a sense of control over their treatment journey (11). This interactivity could range from selecting different areas of the treatment facility to explore, to accessing detailed explanations about each step of the therapy.

In summary, the integration of digital technology in healthcare, particularly through patient-oriented, interactive panoramic virtual tours, can potentially improve education and experience in EBRT. This pilot study aims to describe the general workflow involved with developing a departmental virtual tour and explore its potential for enhancing the patient experience, aligning with the modern trend of patient-centered care in the field of radiation therapy.

## Materials and methods

### Development of the virtual tour

**Figure 1:**
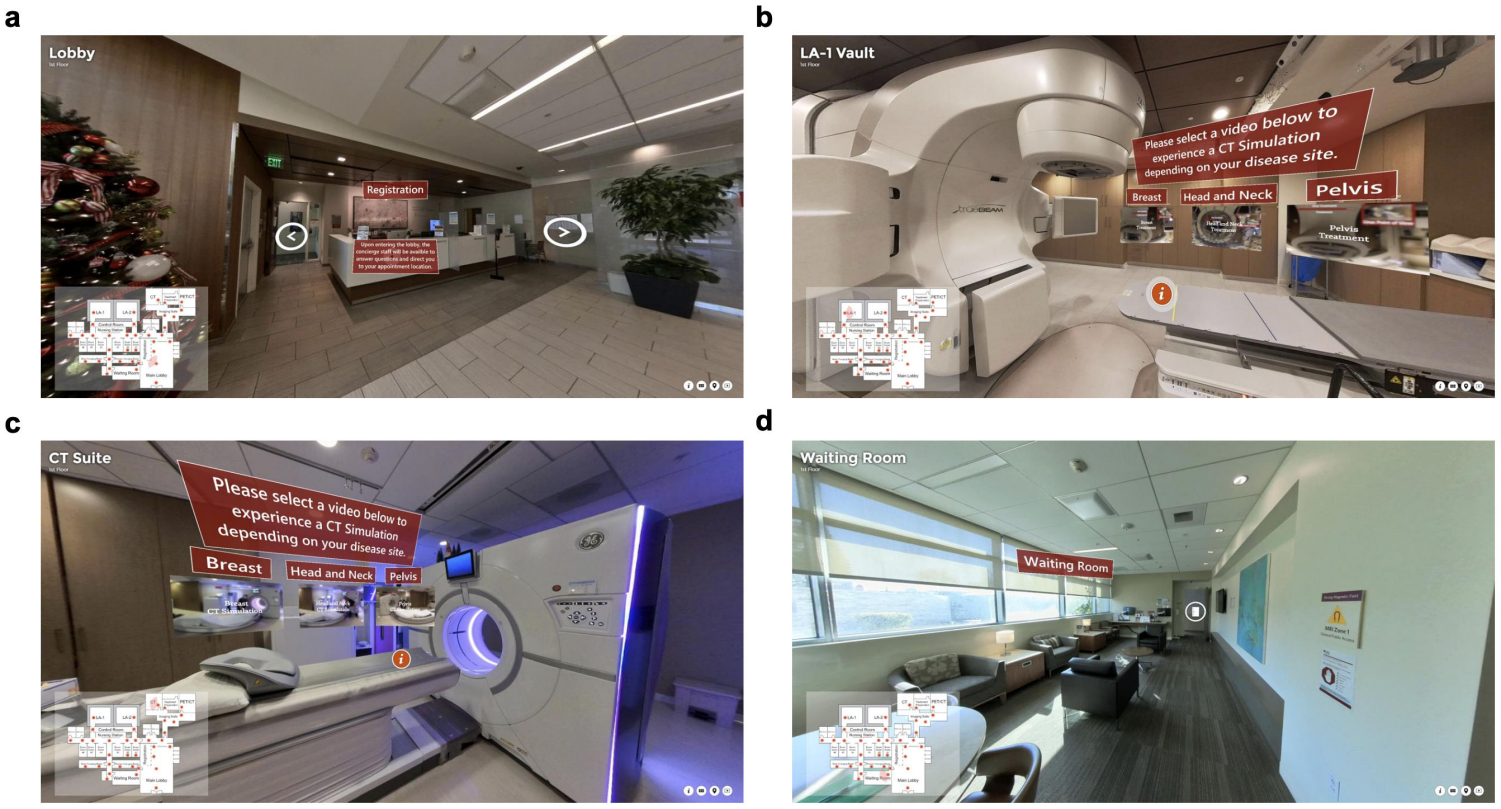
High-resolution panoramic images were captured using the 360-degree 8k camera. These included the **(a)** reception area, **(b)** linear accelerator vault, **(c)** CT simulation suite, and **(d)** waiting room. Previously produced videos, particularly of the 360-degree and Virtual Reality compatible nature were placed at relevant locations throughout the virtual tour for specific disease sites (12). These clips provide a first-person perspective of a patient undergoing simulation and the typical treatment course for breast, head and neck, or pelvic disease sites. Although designed primarily for these sites, all patients could access the videos through the user interface.

**Figure 2:**
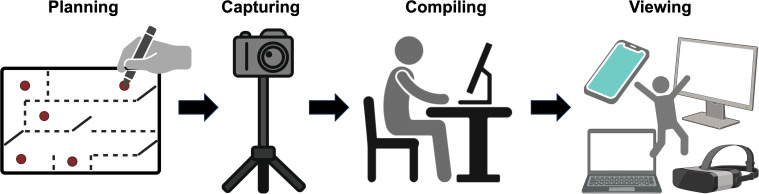
The development process of the virtual tour. The locations of the 360-degree images were planned to encompass the standard care path, then captured. The images were then compiled together using 3D Vista Virtual Tour Pro. When a draft of the virtual tour is completed, it is previewed locally. This process was iterative and usually cycled between compiling new draft versions and viewing. **Figure 2** was created in part with BioRender.com.

**Figure 3:**
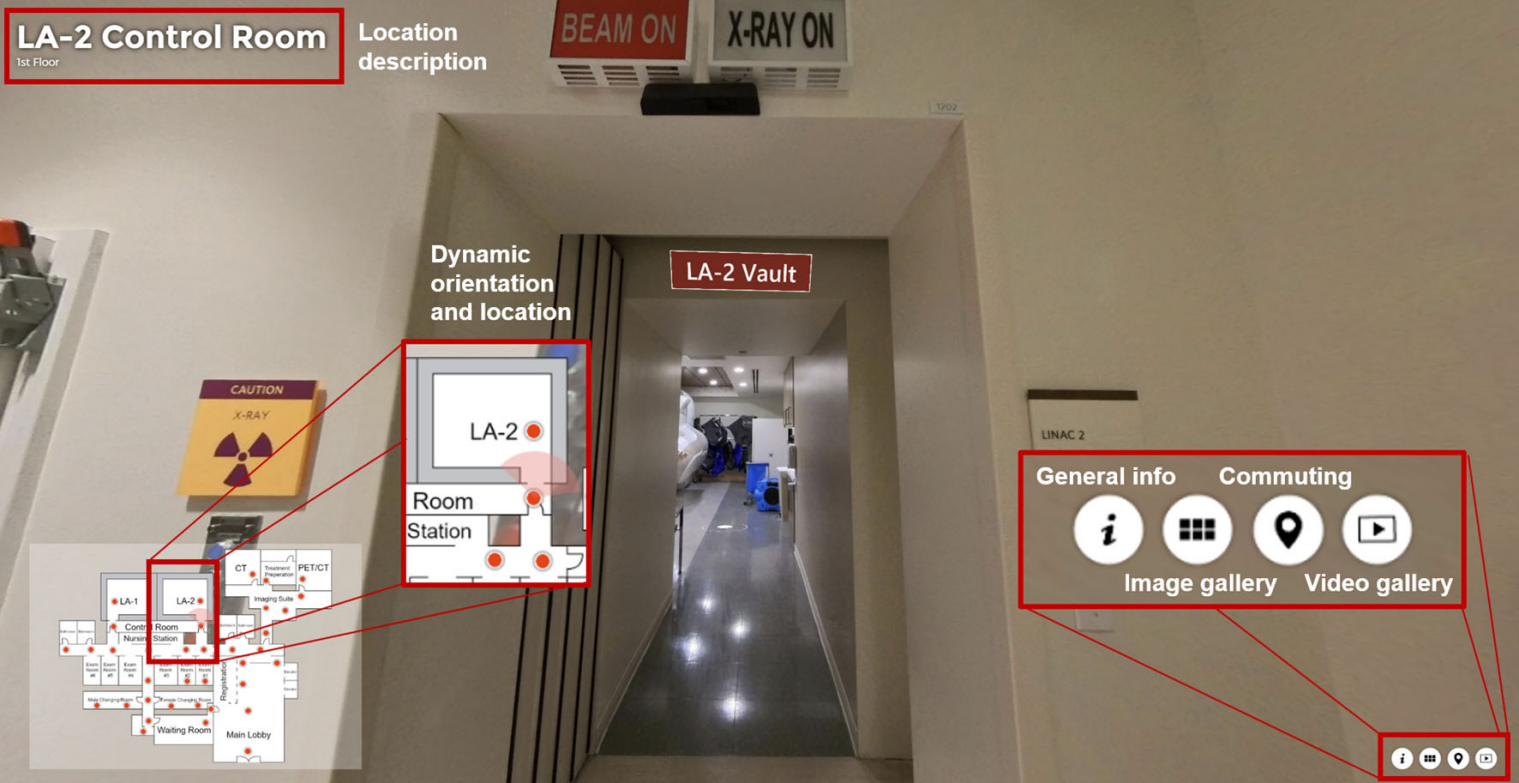
Overview of the user-interface in the virtual tour. A simple map was created to denote the location of all the images, providing a spatial context for the tour. Spots were placed in the map that correspond to the location of the 360-degree picture captured. A cone-shaped-field-of-view allows the viewer to orient themselves. This interface included numerous context pieces, such as a description of the current location, image and video galleries, and digital signage throughout the tour.

The virtual tour was developed using the 3Dvista Virtual Tour Pro software. Critical locations within the radiation oncology department were identified for inclusion in the tour. These included the reception area, waiting rooms, consultation rooms, CT simulation suite, treatment planning room, and the linear accelerator vaults (**Figure 1**). High-resolution panoramic images of these locations were captured using the Insta360 X3 360-degree camera (Insta360, Shenzhen, Guangdong, China). The images were captured at 8k resolution and then compressed for efficient web delivery. This approach ensures that the tour remains visually detailed and informative while being optimized for accessibility and ease of use across various devices. These images were then stitched together using software to create a seamless virtual tour of the department (**Figure 2**). A simple map was also created to denote the location of all the images, providing a spatial context for the facility and tour. Markers were placed in the map that correspond to the location of the 360-degree picture captured. A cone-shaped-field-of-view allows the viewer to orient themselves. A comprehensive user interface was developed to dynamically update and be easily accessible to the patients. This interface included numerous context pieces, such as a description of the current location, and digital signage throughout the tour (**Figure 3**). Approximately 10 minute, 360-degree, Virtual Reality-compatible videos (12) were originally produced for various disease sites and then segmented into 2- to 3-minute clips, which were placed at relevant points within the virtual tour. These clips provide a first-person perspective of a patient undergoing simulation and the typical treatment course for breast, head and neck, or pelvic disease sites. Although designed primarily for these disease sites, all patients could access the videos through the user interface. The virtual tour was published to an institutional secure domain at the time this study commenced, ensuring that experimental group participants could access the link freely. A finalized version of the tour was then published to the public after the study for anyone to freely access.

### Study design

The pilot study was designed aimed to explore the impact of a virtual tour on patient experiences at our two-vault radiation oncology facility, specifically focusing on new patients scheduled to undergo EBRT. The participant selection criteria included all new EBRT patients at our facility, with the exclusion of patients who were either unable to provide informed consent or did not have access to the necessary technology to view the virtual tour. By adopting this exploratory approach, the study aimed to gather preliminary insights into whether a virtual tour could enhance the patient experience.

**Figure 4:**
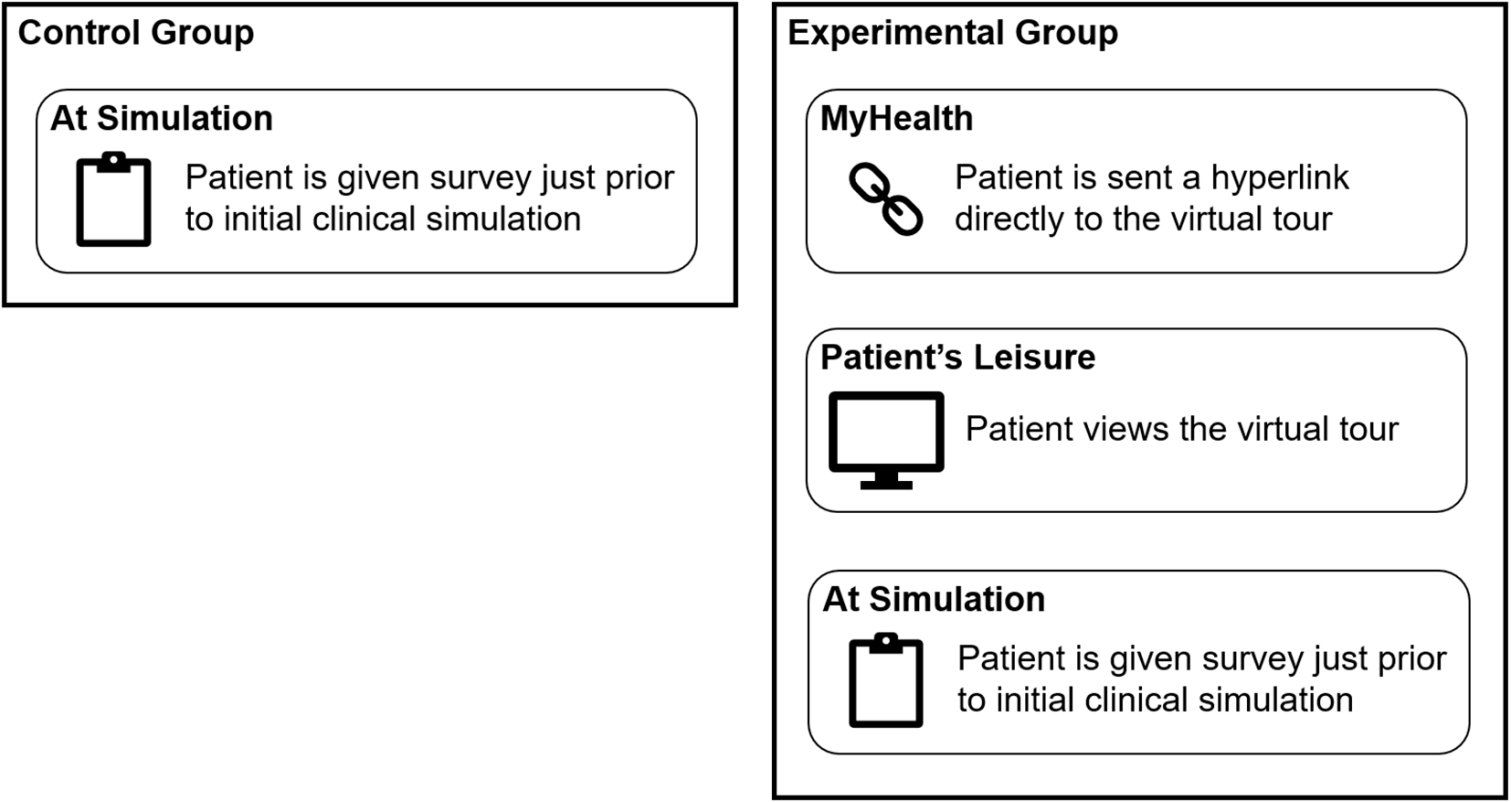
The study design outline of the control and experimental group.

The study was conducted in two distinct groups to compare the experiences of patients who had viewed the virtual tour with those who had not (**Figure 4**). The control group consisted of patients starting a new treatment course, who presented for simulation during the month prior to the virtual tour’s availability, as well as any individuals during the study period who declined or lacked internet access. The survey administered to these patients aimed to assess their levels of anxiety, satisfaction, and comfort, as well as their interest in a virtual reality tour of the facility. This baseline data was critical in establishing the initial emotional and informational state of the patients as they engaged with the facility. The experimental group consisted of patients whose treatment course was initiated during a three-month period after the virtual tour was made available, and who were offered the virtual tour link alongside consultation. During the consultation, each patient in the experimental arm was informed that a virtual tour was available as an optional resource. The nurse coordinator then confirmed whether the patient had an internet-enabled device (computer, tablet, or smartphone). If so, a follow-up message containing a link to the virtual tour was sent via the institution’s secure patient portal, enabling the patient to view the content at their leisure before returning for simulation. In our institution’s workflow, consultations typically occur in a separate clinical area or virtually, so the full simulation suite or treatment vault is not usually seen until the day of simulation. The survey for both groups was administered immediately before the simulation, capturing participants’ baseline impression at a consistent point in the care pathway. The institutional patient care path from consultation to initial clinical simulation was approximately one to two weeks. Following their engagement with the virtual tour, they were surveyed with a set of questions nearly identical to those asked of the control group, also just prior to the initial clinical simulation. This experimental group’s responses were then compared to those of the control group to discern the impact of the virtual tour on patient experiences. All patients, irrespective of study arm, received baseline educational materials routinely provided at our institution. These included a printed brochure outlining the steps of simulation and treatment and access to a basic departmental webpage covering frequently asked questions about radiation therapy.

By employing this two-arm approach, the study aimed to capture an understanding of how the introduction of a virtual tour could influence patient perceptions, anxiety levels, and overall satisfaction within the external beam division of the radiation oncology department.

### Survey instrument

The survey was designed to capture a comprehensive view of the participants’ experiences at the cancer center and their perceptions regarding the utility of a virtual tour (**Supplementary Material**). The survey first contains a binary question about previous visits to the radiation oncology department, followed by a series of 1–10 rating scales. These scales allowed participants to self-report their current anxiety levels, ease of finding their way in the center, satisfaction with the cancer center, current comfort level, and knowledge about the cancer center. The survey also included questions about prior experience with virtual tours of healthcare facilities and interest in viewing a virtual tour before RT. The survey was developed alongside the treating physicians, radiation therapists, and nurse coordinators to ensure relevance and clarity of content.

### Statistical analysis

Descriptive statistics were performed separately for both the control and experimental groups to compare their responses on various survey parameters. Means and standard deviations were calculated for each self-assessment scale. To assess group differences, the Mann-Whitney U test was used for non-parametric comparison of the survey responses, and Levene’s test for equality of variances was applied to examine differences in variability between groups. P-values were reported for both the Mann-Whitney U test (for group differences) and the Levene test (for variance differences). Statistical significance was considered at p < 0.05.

## Results

### Patient population

The study involved 66 participants from a cancer center, divided into a control group (33 out of 38 approached, response rate: 86.8%) and an experimental group (35 out of 97 approached, response rate: 36.1%). The control group in the study consisted of 33 patients, with 39.4% being male, 60.6% female, and an average age of 64.4 years (SD, 14.2 years), with a range in age from 27 to 87 years. In contrast, the experimental group, which viewed the virtual tour, included 35 patients with a slightly higher male representation of 45.7%. The average age in this group was 68.7 years (SD, 12.3 years), spanning from 34 to 86 years. In the control group, 30% of patients reported having visited the radiation oncology department before, while 40% of the experimental group reported having visited before. When asked if they have ever taken a virtual tour of a health care facility before, 12% of the control group reported having viewed, while 18% for the experimental group. When the control group was asked if before their first RT treatment, if they would like to have the option to view a virtual tour, 61% responded yes (**Table 1**).

**Table 1:** Patient characteristics summary.

Patient Characteristics	Control Group (n=33)	Experimental Group (n=35)
Mean Age (SD, Range) [years]	64.4 (14.2, 27:87)	68.7 (12.3, 34:86)
Sex [% Male, % Female]	39.4, 60.6	45.7, 54.3
Visited before? [%]	30	40
Viewed another VT? [%]	12	18
Want to view VT? [%]	61	–

VT, Virtual Tour; SD, Standard Deviation.

### Anxiety, comfort, knowledge, navigation, and satisfaction

**Figure 5:**
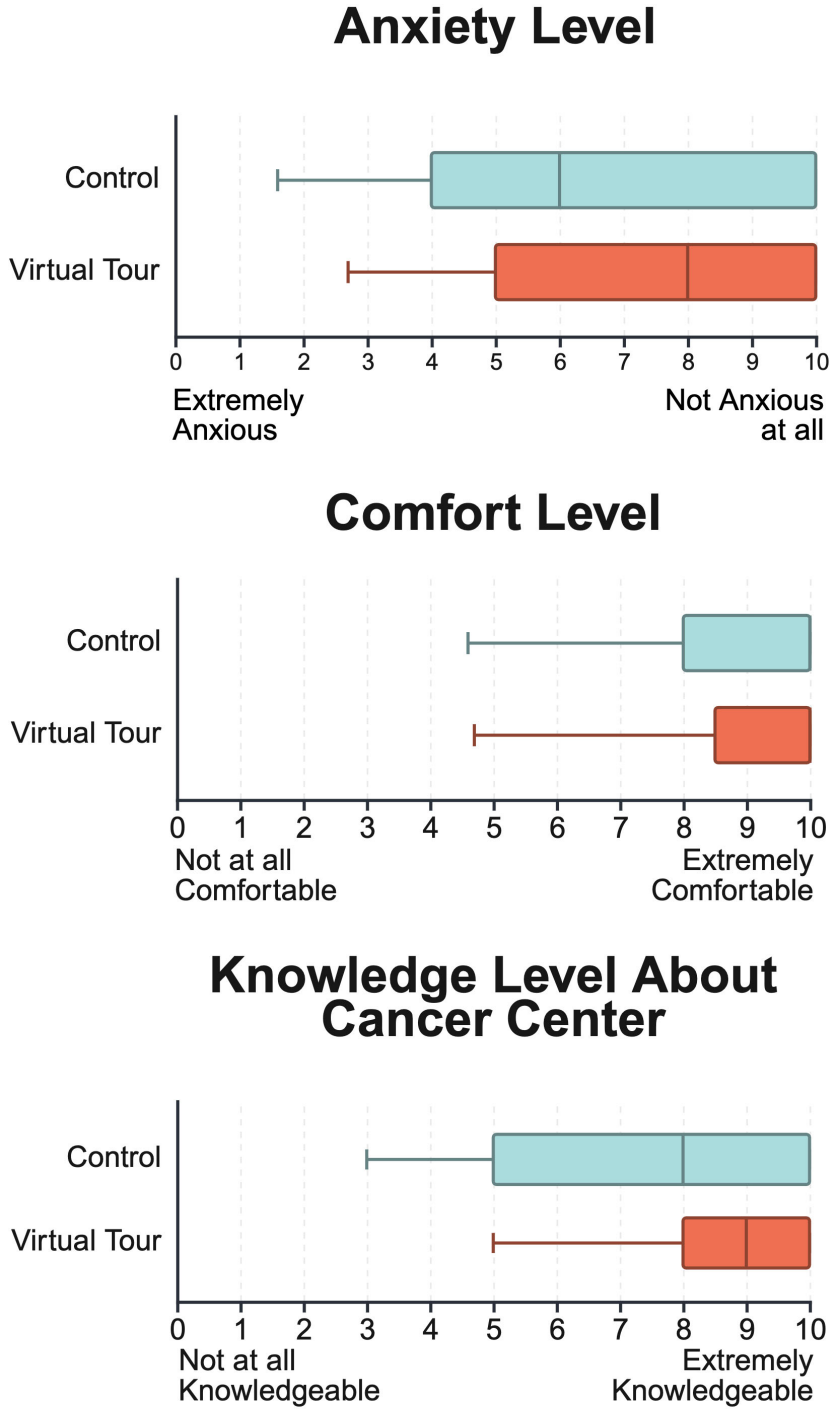
Comparison of anxiety levels, comfort levels, and knowledge about the cancer center between control and virtual tour groups. Scores range from 1 to 10, with higher scores indicating lower anxiety, higher comfort, and greater knowledge. Minimum represents the 5th percentile confidence interval. The virtual tour group showed slightly lower anxiety, marginally higher comfort, and comparable knowledge levels relative to the control group, with tighter score distributions across all measures.

The comparison of responses between the experimental and control groups across several key metrics: anxiety, comfort, knowledge, navigation, and satisfaction are illustrated in **Figure 5**. The mean anxiety level for participants in the experimental group, where a greater score trended to less anxiety, was higher with a mean score of 7.3 (SD = 2.6) compared to the control group’s 6.5 (SD = 3.3). The difference in anxiety levels between the two groups was not statistically significant (p = 0.32), and the standard deviation for anxiety scores was significantly different between the groups (p = 0.03).

For comfort levels, the experimental group’s mean score was 9.0 (SD = 1.8), suggesting a slightly higher comfort level compared to the control group’s mean of 8.4 (SD = 2.2). However, the difference in comfort levels between the two groups was not statistically significant (p = 0.27), and the variability in responses was also similar across both groups (p = 0.21).

When assessing how knowledgeable participants felt about the cancer center, the experimental group’s average score was 8.6 (SD = 1.7), which was higher than the control group’s average of 7.8 (SD = 2.5). The difference in knowledge levels did not reach statistical significance (p = 0.27), and there was a statistically significant difference in the variability of responses between the two groups (p = 0.03).

Regarding the ease with which participants could navigate the cancer center, both groups reported high mean scores. The experimental group’s responses were slightly more favorable, with a mean score of 9.8 (SD = 0.6), compared to the control group’s 9.4 (SD = 2.0). However, the difference in navigation scores between the two groups was not statistically significant (p = 0.61), and the variability in navigation scores was also similar across both groups (p = 0.25).

The satisfaction level with the cancer center revealed similarly high scores from both groups. The experimental group’s mean score was marginally higher at 9.6 (SD = 1.1), compared to the control group’s 9.5 (SD = 1.3). This difference in satisfaction scores was not statistically significant (p = 0.74), and the variability in satisfaction scores was also not significantly different between the two groups (p = 0.85).

### Virtual tour feedback

In the experimental group survey, an open-ended item allowed participants to describe what they found most helpful. The majority (n=11/35) of these brief responses focused on increased familiarity with the facility layout and simpler wayfinding. Of the eleven, five participants mentioned appreciating a clear view of where to check in upon arrival. In addition, five patients (n=5/35) specifically noted the value of seeing the linac or CT scanner in advance. Four patients (n=4/35) appreciated the virtual tour in its entirety. One patient (n=1/35) reported minor technical issues viewing on a smartphone. Four patients (n=4/35) did not find the virtual tour generally helpful. Ten participants (n=10/35) left this question blank and did not provide feedback.

## Discussion

To our knowledge, this pilot study is the first to examine the feasibility and potential impact of a patient-oriented, interactive panoramic virtual tour in EBRT.

### Survey results

The higher mean score for anxiety levels in the experimental group suggests that the intervention, was somewhat associated in reducing anxiety. The lower variability in this group could indicate that the intervention consistently mitigated anxiety across participants. This aligns with existing literature that emphasizes the role of patient education in reducing pre-procedure anxiety (6–8). The slight increase in the mean score for the ease of finding one’s way in the cancer center for the experimental group, coupled with a lower standard deviation, might reflect the tour’s utility in helping patients navigate the physical space of the center. The cancer center where the patients were sampled is a two-vault center, so the improvement in reported navigation may be even greater for larger centers where wayfinding if more complex. The data indicates a small increase in patient satisfaction in the experimental group. The tighter grouping of responses suggests that the intervention uniformly influenced participants’ satisfaction. The comfort level scores from the experimental group not only had a higher mean but also less spread, which could be attributed to the patients feeling more at ease with their surroundings after experiencing the virtual tour. With the highest mean difference between the groups in the measure of how knowledgeable participants felt about the cancer center, the data suggests that the experimental group felt more informed. The lower standard deviation points to a consistent enhancement across individuals in the experimental group. Overall, although the mean improvements did not reach statistical significance for all measures, the standard deviation in scores (as assessed by Levene’s test) was significantly lower for anxiety and knowledge in the experimental group. This suggests a more uniform experience among those who accessed the virtual tour, though it does not establish efficacy without further study with a larger cohort.

The additional questions in the survey gave insightful statistics. Across both the control and experimental groups, approximately 15% of participants had already viewed a virtual tour in a health care setting. This finding highlights a growing familiarity with digital educational tools and may suggest potential for broader adoption across various medical specialties, including radiation oncology. Also, interestingly, among the control groups participants (n=33/38), 61% of control-group patients indicated that they would like to view a virtual tour when surveyed in person, immediately before simulation. However, only 36.1% of the experimental group actually viewed the tour on their own time prior to the simulation and reported this in the survey at their simulation appointment. This discrepancy may be due to the more direct nature of the control group survey, where patients were asked face-to-face about the survey, versus the experimental group option to access the virtual tour independently, potentially competing with other responsibilities or limited digital fluency. In addition, the more indirect process of distributing the virtual tour link during consultation or just after, and the need for later viewing may have decreased motivation or recall. In the experimental group survey, an open-ended item allowed participants to describe what they found most helpful. Overall, feedback was positive, with the majority of patients enjoying either specific parts of the virtual tour or its entirety. A few patients did not find the virtual tour generally helpful. One patient reported minor technical issues viewing on a smartphone, which were addressed soon after.

Overall, the data from the pilot study suggests that the introduction of the interactive virtual tour of the cancer center could be associated with a positive impact on the patient experience, as reflected by slight improvements in self-reported anxiety, ease of navigation, satisfaction, comfort, and knowledge about the center. While these differences did not reach statistical significance for most measures, the significantly reduced variability in anxiety and knowledge scores indicates a more consistent experience among patients who accessed the tour. Such tools could represent an addition to patient care protocols, particularly in preparing patients for the cancer treatment process in larger or more complex facilities. Nonetheless, these findings should be interpreted with caution given the study’s small sample size, lower response rate in the experimental group, and the use of a self-developed, unvalidated survey instrument.

### Limitations

This study has several limitations that affect its generalizability and applicability. First, although the self-developed survey was reviewed by the treating physicians, radiation therapists, and nurse coordinators for content, it was not formally pilot tested or cross-validated. As a result, the instrument’s reliability and validity are not rigorously established, which may impact the consistency of the findings. The small sample size limits the ability to draw broad conclusions, and the study did not control for specific diagnoses or treatments, which could influence the outcomes. Moreover, a greater proportion of participants in the experimental group (40%) than in the control group (30%) had previously visited the radiation oncology department, potentially biasing their reported results. This discrepancy in prior exposure is acknowledged as a potential confounding factor that may have influenced the study’s results.

In addition, the differing response rate between the control (86.6%) and the experimental group (36.1%) may introduce selection bias, limiting the overall generalizability. Despite these limitations, the study offers a promising initial exploration into the effectiveness of virtual tours in RT patient education. Future studies with larger and more diverse patient cohorts, likely at a larger institution, are needed to confirm whether these trends remain consistent and to better quantify the impact of virtual tours on patient-reported outcomes.

In regard to the creation and development of the virtual tour, a key consideration and limitation to overcome was ensuring its accessibility. Drawing inspiration from the principles outlined in studies like those by Madrigal and Le (13), and Schooley et al. (14), we prioritized hosting the tour in an internet browser. This approach aligns with Madrigal and Le’s emphasis on the importance of accessible and well-organized digital media in healthcare, particularly with modern, interoperable standards. By choosing a web-based platform, we ensured that the tour is easily accessible to a wide range of users, regardless of their device type or operating system. To try and maximize the ease of viewing the virtual tour, we were meticulous in ensuring that the virtual tour is not only accessible but also user-friendly and efficient in terms of data usage. To this end, the tour was designed to be lightweight, taking up only about 500 MB in total.

### Challenges and clinical implementation

The clinical implementation begins with communication with the healthcare staff that are heavily involved with the patient experience, such as any wayfinding staff, physicians, nurses and radiation therapists. Integration with the clinic’s existing digital infrastructure is another critical aspect, requiring careful coordination to ensure compatibility with existing tools, such as MyHealth. Technological barriers can pose hurdles, particularly for patients who are not familiar with digital tools, necessitating additional support from the healthcare team. Allocating resources effectively, both in terms of time and budget, is essential for the successful deployment and maintenance of the virtual tour. Ensuring the privacy and security of patient data is another critical concern, especially when integrating the tour with other clinical systems.

### Digital age in radiation therapy for patient education

In the digital age, virtual tours exemplify how digital tools can clarify complex medical procedures, reduce patient anxieties, and enhance knowledge. A virtual tour of the RT process, for instance, helps patients understand what to expect, increasing their sense of control and preparedness. The use of digital mediums in patient education extends from technology-enhanced learning (TEL) to more innovative methods like comics, each offering distinct advantages in educating both patients and healthcare professionals. Kulaksız et al.’s systematic review of TEL in oncology education for medical professionals reveals a variety of digital tools, despite some drawbacks compared to traditional methods (15). This highlights the growing potential of digital mediums in professional training, indirectly benefiting patient education. Marvaso et al. discuss the impact of augmented reality (AR) and virtual reality (VR) in radiation oncology, underscoring their effectiveness in enhancing educational standards and healthcare workers’ skills (16). These technologies also contribute to patient well-being and adherence, showcasing the strength of immersive digital tools in patient education. Sueyoshi et al. explored using comics to educate children about RT, offering a creative approach that simplifies complex medical information into an accessible and engaging format for pediatric patients (17). This diversity in digital mediums reflects the dynamic nature of technological advancements in healthcare. From TEL to creative methods like comics, these tools not only demystify complex medical procedures but also cater to various patient needs. As technology evolves, its role in patient education is poised to grow, providing more personalized and effective educational experiences.

## Conclusion

This pilot study suggests that virtual tours may improve patient experiences in RT. Patients which viewed the virtual tour showed slightly improved, but non-significant, outcomes in comfort, knowledge, and ease of navigation, with notably tighter distributions for anxiety and knowledge about the cancer center compared to the control group. Although the differences were not statistically significant across all measures, the more consistent responses in these areas suggest that introducing virtual tours may be associated with reducing anxiety and enhancing familiarity with the treatment environment. The integration of virtual tours into RT practices reflects a shift towards more interactive and patient-friendly approaches in healthcare by demystifying the RT process and providing accessible information. They represent a step forward in the evolution of patient-centered care, showcasing how technology can be leveraged to meet the needs of patients in a modern healthcare environment. As healthcare continues to evolve, the adoption of such innovative tools will be critical in addressing the challenges and expectations of the 21st-century patient.

## Data Availability

The raw data supporting the conclusions of this article will be made available by the authors, without undue reservation.
