# Supplementary material for: The feasibility of a patient oriented interactive panoramic virtual tour for external beam radiation therapy
Source: Front Oncol. 2025 May 30;15:1568405. doi: 10.3389/fonc.2025.1568405 (PMC12162308; doi:10.3389/fonc.2025.1568405)
Supplement: Supplementary file 2 [file DataSheet2.pdf]

Virtual Tour Survey

Did you view the virtual tour prior to this appointment?	◯ Yes<br>◯ No
If yes, what part of the virtual tour did you find the most helpful?	______________________
Have you visited the radiation oncology department at this cancer center before?	◯ Yes<br>◯ No
Please rate from 1-10 your anxiety level right now. (1 = extremely anxious, 10 = not anxious at all)	______________________
Please rate from 1-10 how easy it was for you to find your way in the cancer center. (1 = not at all easy, 10 = extremely easy)	______________________
Please rate from 1-10 your satisfaction level with the cancer center right now. (1 = not at all satisfied, 10 = extremely satisfied)	______________________
Please rate from 1-10 your comfort level right now. (1 = not at all comfortable, 10 = extremely comfortable)	______________________
Please rate from 1-10 how knowledgeable you feel about the cancer center. (1 = not at all knowledgeable, 10 = extremely knowledgeable)	______________________
Have you ever taken a virtual tour of another health care facility before?	◯ Yes<br>◯ No
